# Role of thioredoxin reductase (TrxB) in oxidative stress response of *Francisella tularensis* live vaccine strain

**DOI:** 10.1128/jb.00173-25

**Published:** 2025-09-03

**Authors:** Matthew Higgs, Zhuo Ma, Anthony Centone, Chandra Shekhar Bakshi, Meenakshi Malik

**Affiliations:** 1 Department of Life Sciences, Albany College of Pharmacy and Health Sciences 1091, Albany, New York, USA; 2 Department of Pathology, Microbiology and Immunology, New York Medical College 8137, Valhalla, New York, USA; University of Florida, Gainesville, Florida, USA

**Keywords:** auranofin, thioredoxin reductase, antibiotic resistance, intramacrophage survival, oxidative stress, *Francisella*

## Abstract

*Francisella tularensis* is an important human pathogen responsible for causing tularemia in the Northern Hemisphere. *Francisella* has been developed as a biological weapon in the past due to its extremely high virulence. *F. tularensis* is a gram-negative, intracellular pathogen that primarily infects macrophages. *F. tularensis* encodes a repertoire of antioxidant enzymes to counteract the reactive oxygen and nitrogen species (ROS/RNS) produced by macrophages in response to infection. Among these, the thioredoxin system is critical for maintaining cellular redox homeostasis by regulating the balance between oxidation and reduction within bacterial cells. This system includes thioredoxins, thioredoxin reductase, and NADPH. Despite its potential importance, the thioredoxin system of *F. tularensis* remains understudied. *F. tularensis* live vaccine strain (LVS) possesses two thioredoxin genes, *trxA1* (*FTL_0611*) and *trxA2* (*FTL_1224*), and a single thioredoxin reductase gene, *trxB* (*FTL_1571*). In this study, we characterized the role of *trxB* of *F. tularensis* LVS in oxidative stress resistance. Our findings demonstrate that *trxB* is essential for oxidative stress resistance in *F. tularensis* and that its loss increases susceptibility to several antibiotics. However, unlike other bacterial species, TrxB in *F. tularensis* is not a functional target of the gold-containing antimicrobial agent auranofin. We also show that OxyR, the master regulator of oxidative stress responses, directly controls *trxB* expression under oxidative stress conditions. Furthermore, TrxB contributes to intramacrophage survival by enabling the bacterium to withstand ROS-induced oxidative stress. Collectively, this study highlights a critical, previously uncharacterized antioxidant defense mechanism in *F. tularensis* and its importance in oxidative stress resistance and intramacrophage survival.

**IMPORTANCE:**

This study elucidates the function of the *trxB* gene, which encodes a thioredoxin reductase, in overcoming oxidative stress by *Francisella tularensis*. Loss of the *trxB* gene results in enhanced susceptibility to oxidants, diminished intracellular survival, and antibiotic resistance. Unlike other bacterial species, *F. tularensis* TrxB is not a functional target of auranofin, a gold-containing antimicrobial compound, suggesting divergence in thioredoxin system interactions. Furthermore, transcriptional regulation of *trxB* by OxyR in response to oxidative stress highlights an adaptive control mechanism essential to resist oxidative stress. These findings provide a mechanistic understanding of *F. tularensis* antioxidant defenses and their role in intramacrophage survival.

## INTRODUCTION

*Francisella tularensis* is an important human pathogen responsible for causing a fatal disease known as tularemia in the Northern Hemisphere. *F. tularensis* has been developed as a biological weapon in the past because of its extremely high virulence. Based on its potential use as a bioterror agent, it is now classified as a Tier 1 Select Agent by the CDC (1, 2). The human virulent *F. tularensis* strains are classified under *F. tularensis* subspecies *tularensis* (Type A) and *F. tularensis* subspecies *holarctica* (Type B) (3). The highly virulent *F. tularensis* SchuS4 strain belongs to *F. tularensis* subspecies *tularensis*, while the live vaccine strain (LVS) is a derivative of *F. tularensis* subspecies *holarctica* developed in the United States from the Russian strain S15 (4). The clinical presentation of tularemia depends on the route, dose, and infecting strain of *F. tularensis*. Percutaneous exposure results in ulceroglandular and oculoglandular forms of tularemia. Pneumonic tularemia is a highly acute form of the disease. The mortality rate due to pneumonic tularemia may be as high as 30–60% in untreated cases (5–7). Tularemia is a notifiable disease in the United States. Naturally occurring tularemia is reported from all the states of the United States, except Hawaii (8, 9). The incidence of tularemia has been on the rise in the past few years in the South-Central, the Pacific Northwest, and Massachusetts, including Martha’s Vineyard (10, 11). No vaccine is currently available for tularemia prophylaxis.

*F. tularensis* is a gram-negative intracellular pathogen that primarily infects macrophages. *F. tularensis* has a unique intramacrophage lifestyle, which includes a transient phagosomal phase, followed by its escape into the cytosol, where bacterial replication occurs. *F. tularensis* is exposed to oxidative stress at both intracellular locations (12). To counteract the oxidative and nitrosative stresses imposed by reactive oxygen and nitrogen species (ROS and RNS) generated by infected macrophages, *F. tularensis* encodes a repertoire of antioxidant enzymes that neutralize these reactive species. Functional studies involving a point mutant of the iron-containing superoxide dismutase B (*sodB*) gene, as well as gene deletion mutants of copper-zinc-containing *sodC* (Δ*sodC* and *sodB*Δ*sodC*), alkyl hydroperoxide reductase (Δ*ahpC*), catalase (Δ*katG*), and the oxidative stress response regulator OxyR (Δ*oxyR*), have demonstrated that these mutants in the *F. tularensis* LVS strain exhibit enhanced sensitivity to ROS and RNS. Moreover, these mutants are significantly attenuated for virulence. In mice, demonstrating a critical role of these antioxidant enzymes as essential virulence factors in *F. tularensis* pathogenesis (13–17). A transmembrane component of the major facilitator superfamily type of efflux pump, EmrA1, confers resistance to oxidants by secreting antioxidant enzymes SodB and KatG (18). Additionally, the outer membrane component SilC of the Emr multidrug efflux pump also confers resistance against oxidative stress (19). The stringent response in *F. tularensis* LVS governs the oxidative stress response by regulating the expression of genes encoded on the Francisella pathogenicity island*,* transcriptional regulators, and antioxidant enzyme genes (20). A transcriptional regulator belonging to the AraC/XylS family also modulates the oxidative stress response of *F. tularensis* LVS (21).

The thioredoxin system of *F. tularensis*, required to mitigate oxidative stress encountered during its intracellular lifecycle, remains understudied. The thioredoxin system is essential for cellular redox regulation and maintaining the balance between oxidation and reduction within bacterial cells. The thioredoxin system is comprised of thioredoxins, thioredoxin reductase (TrxR), and NADPH and protects bacteria from oxidative stress by regulating DNA synthesis, repair, and apoptosis (22). Thioredoxins are small proteins that serve as key antioxidants, primarily reducing other proteins through cysteine thiol-disulfide exchange. In this process, thioredoxins themselves become oxidized, forming a disulfide bond between their active-site cysteines. TrxR regenerates thioredoxins to their reduced form using electrons from NADPH. This system in *Francisella* includes two thioredoxins, TrxA1 and TrxA2, and a single TrxR annotated as TrxB. TrxA1 regulates the oxidative stress response of *F. tularensis* LVS by modulating the expression of the master regulator of oxidative stress, OxyR. However, unlike TrxA2, TrxA1 is essential for resisting oxidative stress and survival within macrophages, highlighting its unique mechanism of oxidative stress response regulation in *Francisella* compared to other bacterial thioredoxins (23). In this study, we characterized the role of TrxB in the oxidative stress resistance of *F. tularensis* LVS.

## RESULTS

### Confirmation of *trxB* gene deletion, transcomplementation, and bioinformatic analysis

The *trxB* (*FTL_1571*) gene in *F. tularensis* LVS is 951 base pairs long and encodes a protein of 316 amino acids. The amino acid sequences of TrxB of *F. tularensis* LVS show 98.73% homology to TrxB (*FTT_0489*c) of *F. tularensis* SchuS4 and 99.37% homology to TrxB (*FTN_0580*) of *F. novicida* (not shown). Amino acid sequence alignment of TrxB of *Francisella* with TrxRs of *Helicobacter pylori*, *Mycobacterium tuberculosis*, *Pseudomonas aeruginosa*, *Vibrio cholerae*, *Yersinia pestis*, *Escherichia coli*, and *Salmonella* Typhi revealed four conserved motifs essential for protein functions (Fig. S1A). The GXGXXG motif, which serves as a nucleotide-binding domain that enables the binding of the NADPH cofactor, is critical for the reductase activity of the TrxB (24). The conserved CXXC motif (25), located within the active site, facilitates electron transfer from NADPH to thioredoxin. The presence of cysteine residues at both ends of this motif is particularly important for forming disulfide bonds during redox reactions. The GXGXXA motif is required to maintain the structural integrity of the NADPH-binding pocket, ensuring an efficient cofactor interaction. The HRRXXXR motif is involved in protein-protein interactions and contributes to the specificity and functional activity of the TrxB. The conservation of these functional motifs across various bacterial species signifies their conserved role in TrxRs in maintaining redox homeostasis and resistance against oxidative stress.

A gene deletion mutant of the *trxB* gene (Δ*trxB*) of *F. tularensis* LVS was constructed using allelic replacement methods described earlier (26, 27). The deletion of the *trxB* gene in the Δ*trxB* mutant was confirmed by PCR using primers internal to the *trxB* gene. The absence of the *trxB-*specific amplification product, but the presence of a product corresponding to the *sodB* gene of *F. tularensis* that was used as an internal control, confirmed the deletion of the *trxB* gene. A transcomplemented strain of the *F. tularensis* LVS Δ*trxB* mutant was generated by introducing a copy of the *trxB* gene carried on the transcomplementation vector pMP822 (28). This vector utilizes the blaB promoter, derived from the *F. tularensis* β-lactamase gene, to drive the expression of the *trxB* gene (28, 29). Amplification of a *trxB-*specific fragment by PCR in the transcomplemented strain confirmed the transcomplementation (Fig. S1B and C).

### TrxB contributes to the oxidative stress resistance of *F. tularensis*

**Fig 1:**
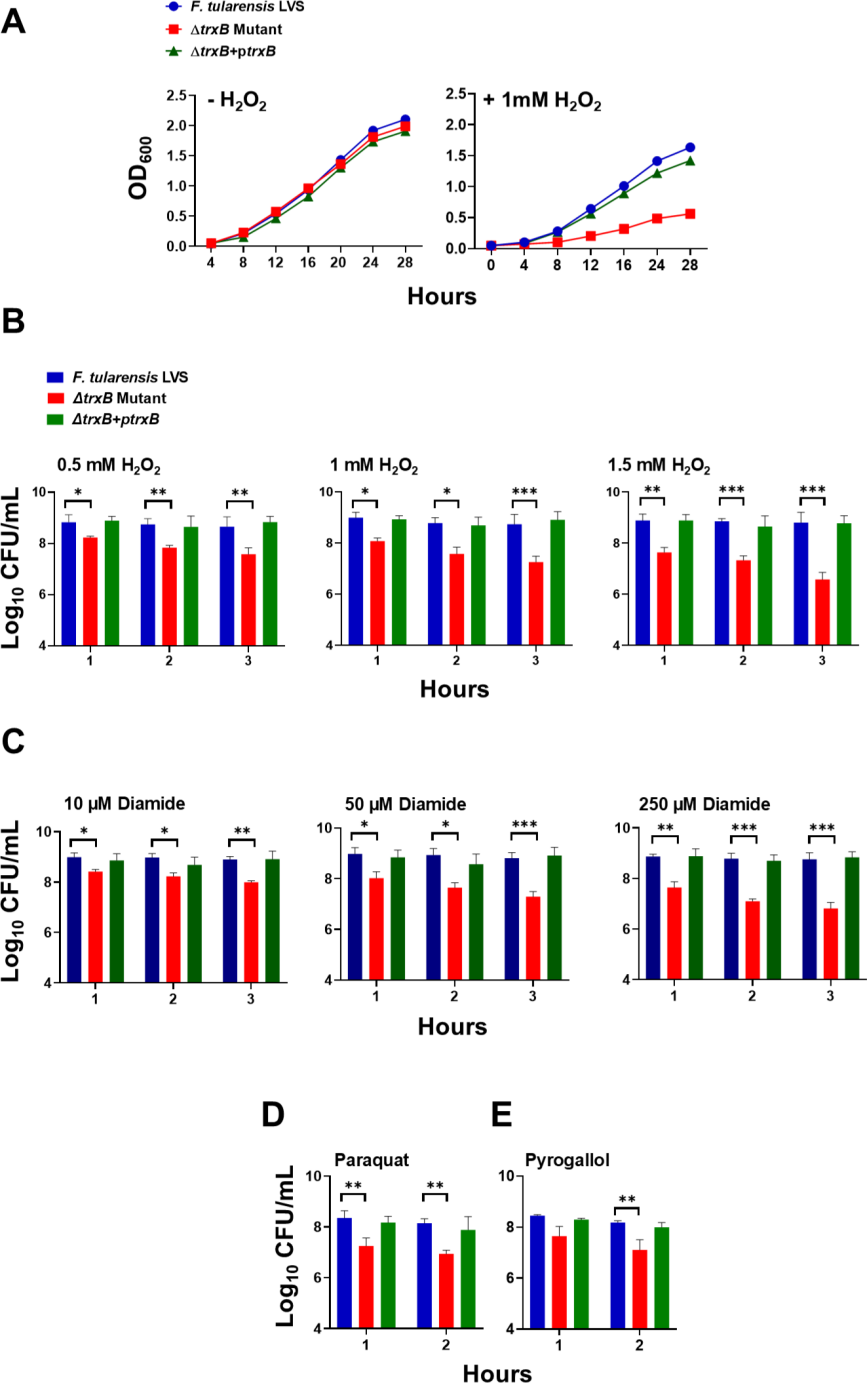
TrxB contributes to the oxidative stress resistance of *F. tularensis*. (**A**) The growth curves of *F. tularensis* LVS, Δ*trxB* mutant, and transcomplemented strain (Δ*trxB* + p*trxB*) in the absence or the presence of 1 mM hydrogen peroxide (H_2_O_2_). (**B–E**) Bacterial killing assay of the *F. tularensis* LVS, Δ*trxB* mutant, and transcomplemented strain (Δ*trxB* + p*trxB*) in the presence of indicated concentrations of H_2_O_2_ (**B**), diamide (**C**), paraquat (1 mM) (**D**), and pyrogallol (1 mM) (**E**) at the indicated times post-exposure. The data shown in (A) are representative of three independent experiments with similar results. The data in (B–E) are presented as colony-forming units (CFU) per mL and represent the cumulative results of three independent experiments, each conducted with three technical replicates. Values are shown as mean ± SEM. The data were analyzed using one-way ANOVA. *P < 0.05, **P < 0.01, and ***P < 0.001.

The growth of *F. tularensis* LVS, the Δ*trxB* mutant, and the transcomplemented strain was assessed under untreated and H_2_O_2_-treated conditions by monitoring OD_600_ values over time. In untreated conditions, the wild-type (WT) *F. tularensis* LVS, the Δ*trxB* mutant, and the transcomplemented strain demonstrated normal growth rates and no growth defects. However, when exposed to H_2_O_2_, the WT *F. tularensis* LVS exhibited a slower growth rate than in untreated conditions, yet still showed a gradual increase in OD_600_ values over time. In contrast, the Δ*trxB* mutant displayed minimal growth following H_2_O_2_ treatment, suggesting an enhanced susceptibility to oxidative stress relative to the WT *F. tularensis* LVS. The transcomplemented strain showed improved growth compared to the Δ*trxB* mutant, although the OD_600_ values did not reach the levels observed for the WT *F. tularensis* LVS (Fig. 1A).

The sensitivity of WT *F. tularensis* LVS, the *∆trxB* mutant, and the trans-complemented strain to oxidative stress was further evaluated by exposing the bacteria to 0.5, 1, and 1.5 mM H_2_O_2_, or 10, 50, and 250 µM of diamide for 1, 2, and 3 h, and 1 mM paraquat and pyrogallol for 1 and 2 h. Across all conditions and time points, the *∆trxB* mutant exhibited reduced viability as indicated by significantly lower colony-forming units (CFUs) compared to the WT *F. tularensis* LVS and the transcomplemented strain, indicating its increased susceptibility to oxidative stress caused by these oxidants (Fig. 1B through E). These findings demonstrate that the *trxB* gene is required for oxidative stress resistance in *F. tularensis*.

### The Δ*trxB* mutant exhibits enhanced sensitivity to several antibiotics

Antibiotics such as ciprofloxacin, levofloxacin, and nalidixic acid exert their bactericidal effects, at least in part, by triggering the Fenton reaction, which generates ROS such as hydroxyl radicals (30). Based on this, we hypothesized that the deletion of *trxB* may increase the susceptibility of the Δ*trxB* mutant to these ROS-generating antibiotics. To test this hypothesis, we conducted antibiotic sensitivity assays using ciprofloxacin, levofloxacin, and nalidixic acid, alongside other antibiotics whose primary mechanisms of action are independent of oxidative stress. Antibiotic susceptibility of the Δ*trxB* mutant was compared to the WT *F. tularensis* LVS and the transcomplemented strain using a conventional disk diffusion assay. No differences in susceptibility between the Δ*trxB* mutant, the WT *F. tularensis* LVS, and the transcomplemented strain were observed for ampicillin, carbenicillin, streptomycin, erythromycin, gentamycin, chloramphenicol, and tetracycline. For ciprofloxacin and levofloxacin, the Δ*trxB* mutant exhibited significantly enhanced sensitivity compared to the WT and the transcomplemented strain. For nitrofurantoin, the WT and the transcomplemented strains showed similar levels of susceptibility, whereas the Δ*trxB* mutant displayed a reduced zone of inhibition, indicating decreased sensitivity. Nalidixic acid exposure revealed a greater zone of inhibition for the Δ*trxB* mutant compared to the WT *F. tularensis* LVS and the transcomplemented strain, indicating statistically significant enhanced susceptibility in the mutant (Table 1). These results demonstrate that *trxB* gene deletion in *F. tularensis* LVS increases susceptibility to ROS-generating antibiotics, indicating a role of TrxB in resistance against oxidative stress-inducing antibiotics.

**TABLE 1:** Antibiotic susceptibility of the Δ*trxB* mutant of *F. tularensis* LVS

Antibiotic	Concentration (µg/per disk)	*F. tularensis* LVS	Δ*trxB* mutant	Δ*trxB* + p*trxB*
Ampicillin	2	6 ± 0^*a*^	6 ± 0	6 ± 0
Carbenicillin	100	6 ± 0	6 ± 0	6 ± 0
Streptomycin	10	25.66 ± 0.51	25.42 ± 0.57	25.62 ± 0.64
**Ciprofloxacin^*b*^**	5	6 ± 0	**9.13 ± 0.31**	6 ± 0
**Levofloxacin**	5	6 ± 0	**8.27 ± 0.50**	6 ± 0
**Nitrofurantoin**	100	35.17 ± 1.69	**28.14 ± 3.06**	35.58 ± 1.26
**Nalidixic acid**	30	30.38 ± 1.11	**39.66 ± 0.88**	31.00 ± 1.37
Chloramphenicol	30	36.83 ± 1.10	36.41 ± 0.91	36.69 ± 1.85
Tetracycline	30	30.51 ± 1.41	30.13 ± 2.06	30.68 ± 1.12
Gentamycin	10	31.13 ± 0.41	31.36 ± 1.75	31.16 ± 1.31
Erythromycin	15	6 ± 0	6 ± 0	6 ± 0

^*a*^ Zone of inhibition in millimeters (mm), including the 6 mm diameter of the antibiotic discs.

^*b*^ Antibiotics shown in bold letters exhibited significantly larger zones of inhibition than wild-type *F. tularensis* LVS and the transcomplemented strain (*P* < 0.05, one-way ANOVA).

### The Δ*trxB* mutant of *F. tularensis* does not exhibit enhanced sensitivity to auranofin

**Fig 2:**
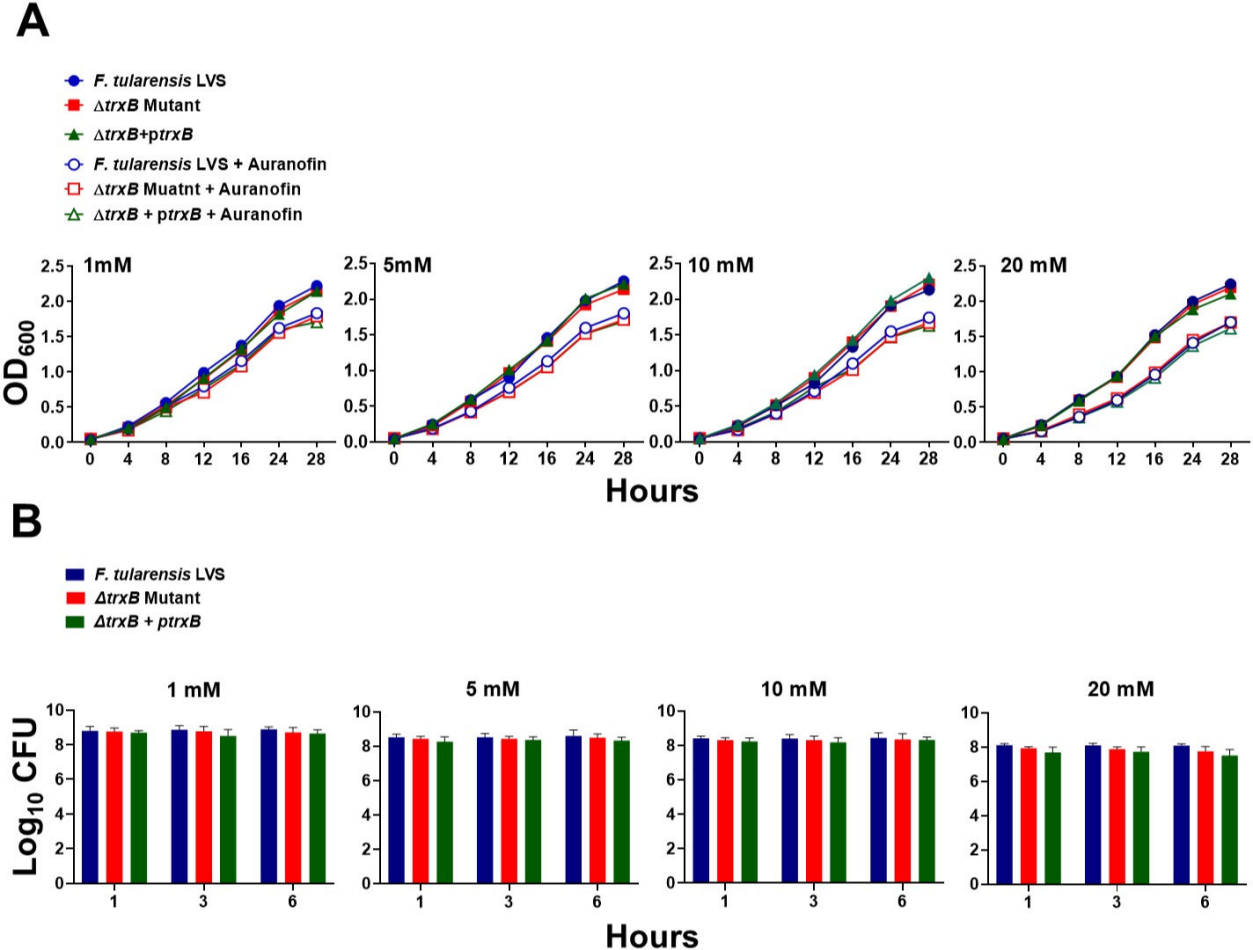
The Δ*trxB* mutant of *F. tularensis* does not exhibit enhanced sensitivity to auranofin. The growth curves (**A**) and bacterial killing assays (**B**) of *F. tularensis* LVS, Δ*trxB* mutant, and transcomplemented strain (Δ*trxB* + p*trxB*) in the absence or the presence of indicated concentrations of auranofin. The data shown in (A) are representative of three independent experiments with similar results. The data in (B) are presented as colony-forming units (CFUs) per mL and represent the cumulative results of three independent experiments, each conducted with three technical replicates, and are shown as mean ± SEM. The data were analyzed using one-way ANOVA.

Auranofin, a gold-containing compound, is known to specifically target the TrxR enzymes in bacteria, inhibiting their activity. Previous studies have demonstrated that bacterial strains lacking TrxR exhibit resistance to auranofin (31, 32). We investigated whether TrxB serves as a target of auranofin in *F. tularensis* by performing growth curve and bacterial killing assays. Exposure to increasing concentrations of auranofin resulted in reduced bacterial growth across WT *F. tularensis* LVS, the *∆trxB* mutant, and the transcomplemented strain compared to untreated controls (Fig. 2A). Moreover, identical numbers of viable bacteria were recovered for all three strains at 1, 3, and 6 h following exposure to increasing concentrations of auranofin (Fig. 2B). These findings indicate that, in contrast to other bacterial species, the TrxB in *F. tularensis* is not a functional target of auranofin.

### Expression of oxidative stress response genes and proteins is decreased in the Δ*trxB* mutant when exposed to oxidative stress caused by H_2_O_2_ and diamide

**Fig 3:**
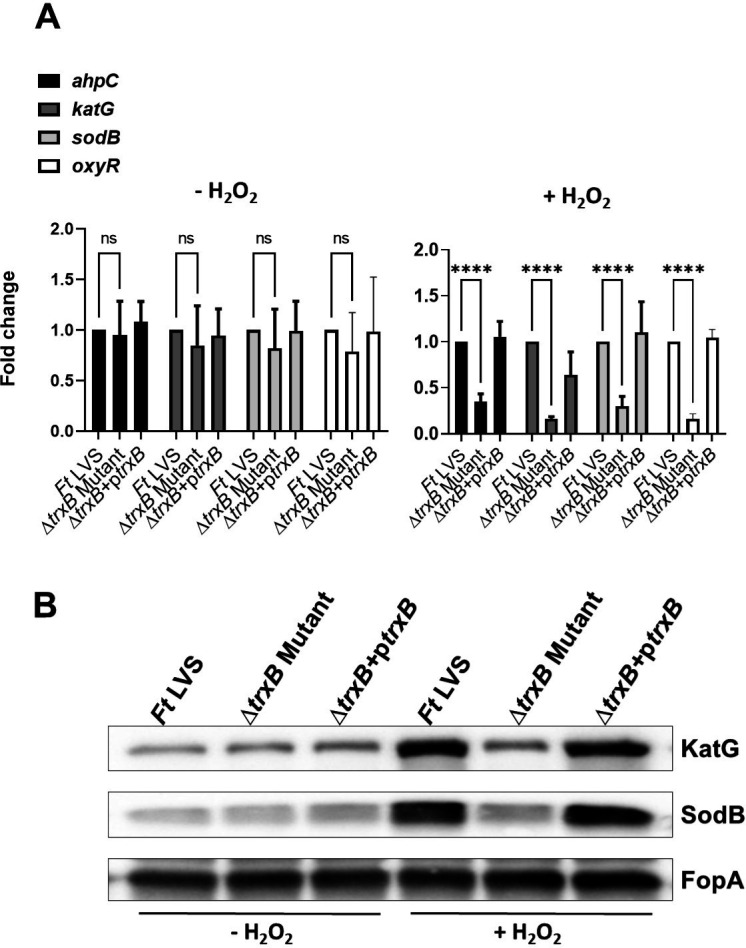
Expression of oxidative stress response genes and proteins is decreased in the Δ*trxB* mutant when exposed to oxidative stress caused by H_2_O_2_. (**A**) Wild-type *F. tularensis* LVS, Δ*trxB* mutant, and the transcomplemented strain (Δ*trxB* + p*trxB*) were either left untreated or exposed to 1 mM H_2_O_2_ for 2 h to induce oxidative stress. The RNA was isolated, and the expression levels of *ahpC*, *katG*, *sodB*, and *oxyR* genes were quantified by qRT-PCR. The data are represented as the relative fold change compared to wild-type *F. tularensis* LVS. The data shown represent the cumulative results of three independent experiments, each conducted with three technical replicates, expressed as mean ± SEM, and analyzed using one-way ANOVA. ****P < 0.0001. (**B**) The western blot analysis of the lysates of the indicated *Francisella* strains in the absence or the presence of 1 mM H_2_O_2_ probed with anti-*Francisella* KatG antibodies. The blots were stripped and re-probed with anti-*Francisella* sodB antibodies. The blots were stripped again and re-probed with anti-*Francisella* FopA antibodies that served as loading controls. The results are representative of two independent experiments conducted.

**Fig 4:**
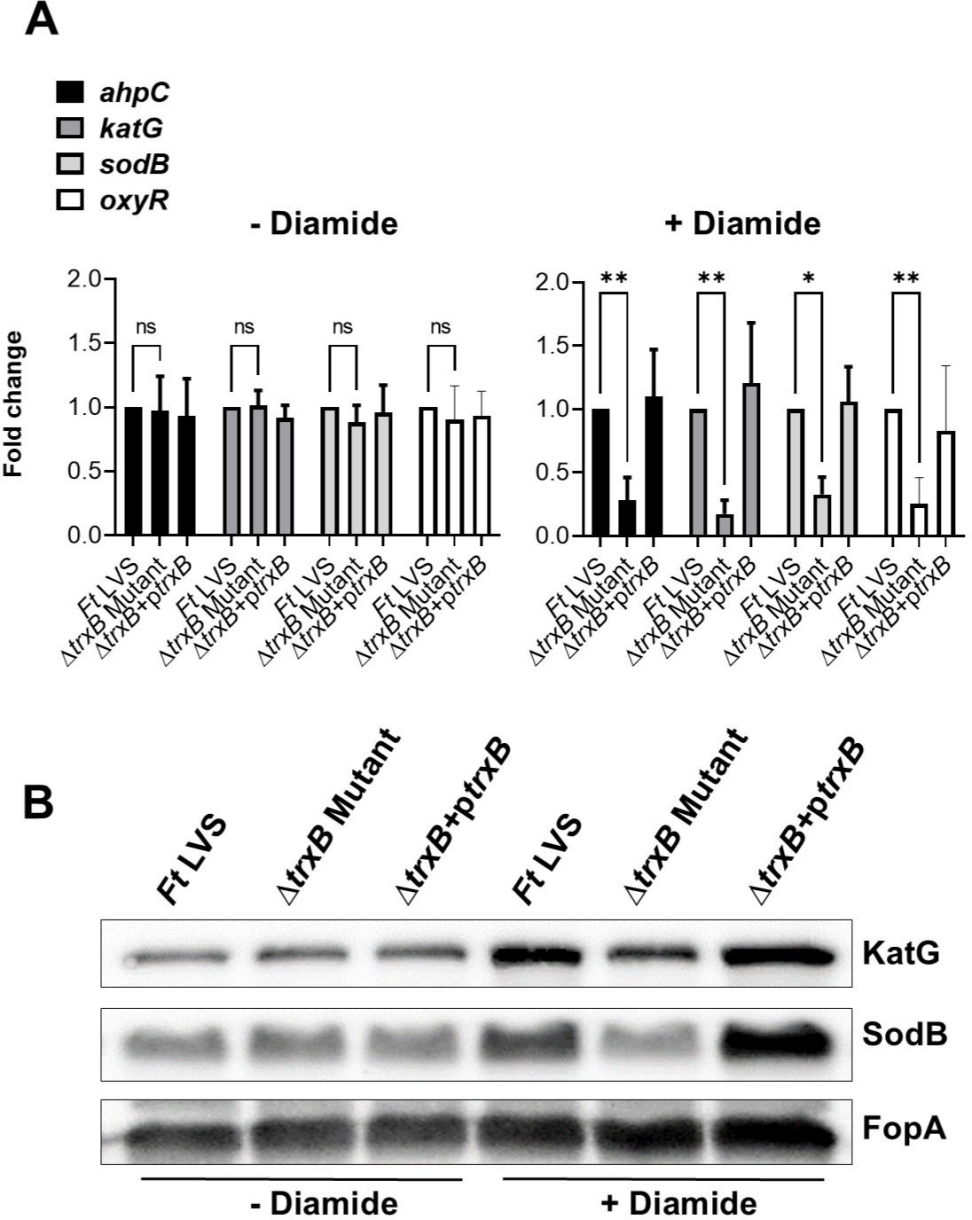
Expression of oxidative stress response genes and proteins is downregulated in the Δ*trxB* mutant when exposed to oxidative stress caused by diamide. (**A**) Wild-type *F. tularensis* LVS, Δ*trxB* mutant, and the transcomplemented strain (Δ*trxB* + p*trxB*) were either left untreated or exposed to 50 µM of diamide for 2 h to induce oxidative stress. The RNA was isolated, and the expression levels of *ahpC*, *katG*, *sodB*, and *oxyR* genes were quantified by qRT-PCR. The data are represented as the relative fold change compared to wild-type *F. tularensis* LVS. The data represent the cumulative results of three independent experiments, each conducted with three technical replicates, expressed as mean ± SEM, and analyzed using one-way ANOVA. *P < 0.05 and **P < 0.01. (**B**) The western blot analysis of the lysates of the indicated *Francisella* strains in the absence or the presence of 50 µM of diamide probed with anti-*Francisella* KatG antibodies. The blots were stripped and re-probed with anti-*Francisella* sodB antibodies. The blots were stripped again and re-probed with anti-*Francisella* FopA antibodies that served as loading controls. The results are representative of two independent experiments conducted.

We next investigated the role of TrxB in the oxidative stress response of *F. tularensis* upon exposure to oxidative stress inducers H_2_O_2_ and diamide. The expression profiles of the oxidative stress response genes alkyl hydroperoxyreductase C (*ahpC*)*,* catalase (*katG*)*,* and superoxide dismutase B (*sodB*), as well as the oxidative stress response regulator gene (*oxyR*), were assessed under both non-stress (−H_2_O_2_) and oxidative stress (+H_2_O_2_) conditions in the WT *F. tularensis* LVS, the Δ*trxB* mutant, and the transcomplemented strains (Fig. 3A). Under non-stress conditions, the expression levels of all genes remained consistent across the WT *F. tularensis* LVS, the Δ*trxB* mutant, and the transcomplemented strains, with no significant differences observed. Following exposure to oxidative stress induced by H_2_O_2_, the expression of primary antioxidant enzyme genes *ahpC*, *katG*, *sodB*, and *oxyR* was significantly decreased in the Δ*trxB* mutant compared to the WT *F. tularensis* LVS and the transcomplemented strain (Fig. 3A). Western blot analysis corroborated these findings, showing markedly reduced levels of KatG and SodB proteins in the Δ*trxB* mutant relative to the WT *F. tularensis* LVS or the transcomplemented strain. FopA was used as a loading control and was demonstrated uniform expression across all conditions (Fig. 3B). Identical results were observed in response to the oxidative stress induced by diamide (Fig. 4A and B). Collectively, these findings highlight the critical role of TrxB in regulating the oxidative stress response in *F. tularensis* LVS and demonstrate that its loss is associated with reduced expression of antioxidant enzyme genes, leading to an enhanced sensitivity to oxidative stress.

### The expression of TrxB is regulated by OxyR in *F. tularensis* LVS

**Fig 5:**
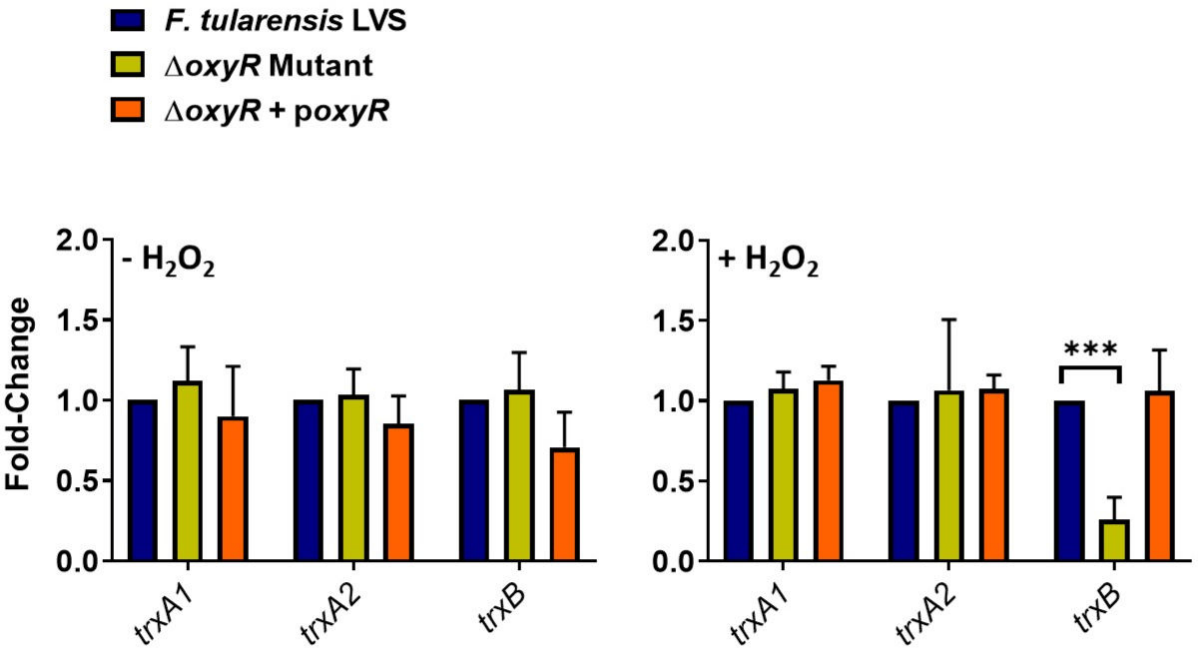
The expression TrxB is regulated by OxyR in *F. tularensis* LVS. Wild-type *F. tularensis* LVS, Δ*oxyR* mutant, and the transcomplemented strain (Δ*oxyR* + p*oxyR*) were either left untreated or exposed to 1 mM H_2_O_2_ for 2 h to induce oxidative stress. The RNA was isolated, and the expression levels of thioredoxin A1 (*trxA1*)*,* thioredoxin A2 (*trxA2*), and thioredoxin reductase (*trxB*) genes were quantified by qRT-PCR. The data are represented as the relative fold change compared to wild-type *F. tularensis* LVS. The data represent cumulative results of three independent experiments, each conducted with three technical replicates, expressed as mean ± SEM, and analyzed using one-way ANOVA. ***P < 0.0001.

Since we observed that the expressions of major OxyR-regulated antioxidant enzyme genes *ahpC, katG,* and *sodB* were decreased in the Δ*trxB* mutant under the conditions of oxidative stress, we next investigated if the *trxB* gene is regulated by OxyR in *F. tularensis* LVS. We determined the expression profile of the thioredoxin genes *trxA1* and *trxA2*, which are not regulated by OxyR (23), along with the *trxB* gene in WT *F. tularensis* LVS, the Δ*oxyR* mutant, and the transcomplemented strain. In the absence of oxidative stress, no differences in the expression of *trxA1, trxA2,* and *trxB* genes were observed. However, the expression of the *trxB* gene was significantly decreased in the Δ*oxyR* mutant, in the presence of oxidative stress induced by H_2_O_2_, while the expression of *trxA1* and *trxA2* genes remained unaltered (Fig. 5). These results indicated that the expression of *trxB* is regulated by OxyR. Furthermore, the decreased expression of *oxyR* in the Δ*trxB* mutant (Fig. 3 and 4), and conversely of *trxB* in the Δ*oxyR* mutant (Fig. 5), suggest a reciprocal regulation of these antioxidant defense mechanisms.

**Fig 6:**
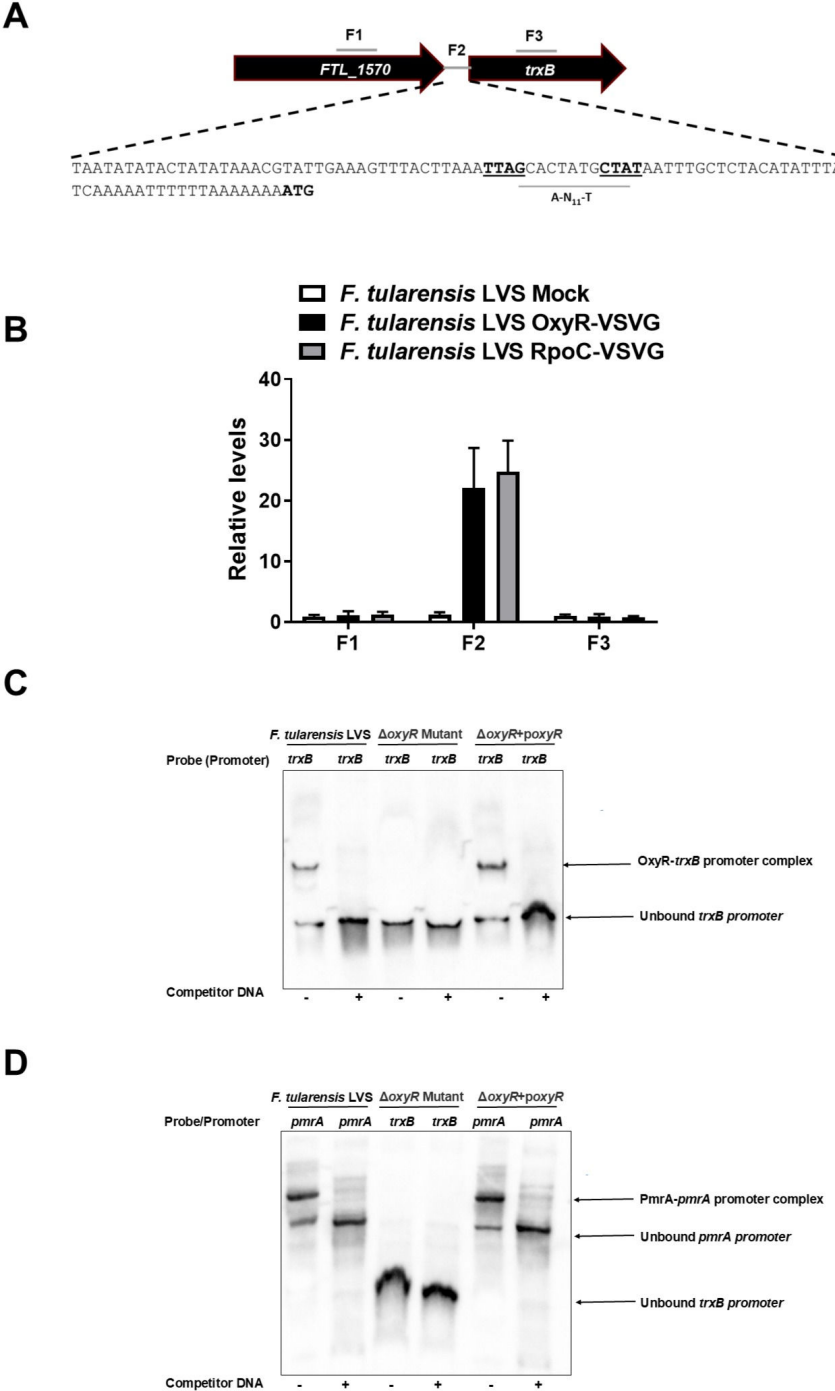
OxyR in *F. tularensis* LVS regulates the expression of TrxB. (**A, B**) Chromatin immunoprecipitation (ChIP) was performed using anti-VSV-G-agarose beads. The fragments of intergenic regions covering the putative promoter sequences and the coding regions of the genes up and downstream of the *trxB* gene (indicated by numbers) in ChIP and input samples of the indicated bacterial strains were analyzed for enrichment by qRT-PCR. The values were normalized to the input and a *fopA* coding region as internal controls. The qRT-PCR results of ChIP from *F. tularensis* LVS OxyR-VSVG, along with wild-type *F. tularensis* LVS (mock) and RpoC-VSVG (positive control) are shown (*n* = 3 technical replicates). The data shown are representative of three independent experiments and were analyzed by ANOVA. (**C**) Electrophoretic mobility shift assay (EMSA) with the promoter region for *trxB* of *F. tularensis*. EMSA was performed using bacterial lysates from the *F. tularensis* LVS, the Δ*oxyR* mutant, and the transcomplemented strain (Δ*oxyR* + p*oxyR*). The biotinylated DNA sequence of the promoter region was used as the probe, whereas the unlabeled promoter region was used as competitor DNA. (**D**) EMSA with the promoter region for the *pmrA* gene of *F. tularensis*. The activity of lysates from the Δ*oxyR* mutant was determined by binding of transcriptional regulator PmrA to its putative *pmrA* promoter region by EMSA. A biotinylated 505 bp fragment of the *pmrA* promoter region was used as a probe, whereas an unlabeled promoter region was used as competitor DNA.

We further investigated how OxyR, the master regulator of oxidative stress, regulates the expression of *trxB* in *F. tularensis* LVS. We investigated if OxyR regulates the expression of *trxB* by binding directly to the promoter region of *trxB* by chromatin immunoprecipitation (ChIP) and electrophoretic mobility shift assay (EMSA) analyses. The *trxB* promoter region was analyzed for an OxyR binding region. To investigate the binding activity of OxyR to the *trxB* promoter region, an *F. tularensis* LVS strain expressing OxyR fused to a C-terminal Vesicular Stomatitis Virus Glycoprotein (VSVG) tag was constructed, as previously described (16). This epitope-tagged OxyR-VSVG strain enables ChIP assays using anti-VSVG agarose beads to detect specific DNA-protein interactions. As a positive control for promoter activity, the tagging integration vector pKL02, encoding an RpoC-VSVG fusion protein, was used. In this strain, the β′ subunit of RNA polymerase is tagged with VSVG, allowing ChIP-based detection of RNA polymerase binding to active promoter regions. This control not only confirms transcriptional engagement at the promoter but also ensures that observed transcription factor binding corresponds to a transcriptionally competent site (33). Binding sites upstream of the *trxB* gene (F1), within the intergenic region between *FTL_1570* and *trxB* (F2), and within the *trxB* coding sequence (F3) were tested for OxyR interaction. ChIP assays revealed nearly 20-fold enrichment of the F2 region, which is immediately upstream of *trxB* and contains an A-N_11_-T OxyR-binding domain indicating significant OxyR binding to the *trxB* promoter within the F2 intergenic region (Fig. 6A). No OxyR-VSVG binding was observed in F1 or F3, nor was any binding detected in the mock controls across F1, F2, and F3 regions, confirming the direct interaction of OxyR with the *trxB* promoter. The *F. tularensis* LVS RpoC-VSVG strain included as an additional control exhibited enrichment exclusively in the F2 region, with no binding detected in F1 or F3 regions (Fig. 6B). The enrichment of the intergenic F2 site in the RpoC-VSVG strain further supports the conclusion that OxyR binding is specific to a functionally relevant promoter region. Taken together, these results provide strong evidence that OxyR directly regulates *trxB* through defined promoter region interactions.

To validate and further explore OxyR binding specificity, EMSA assays were conducted using the *trxB* gene promoter probes. In the WT *F. tularensis* LVS, OxyR bound specifically to the *trxB* promoter probe, an interaction that was disrupted upon the addition of competitor DNA, confirming its sequence specificity. Binding was notably absent in the Δ*oxyR* mutant strain, whereas the Δ*oxyR* + p*oxyR* transcomplemented strain restored the OxyR-*trxB* promoter binding (Fig. 6C). As an additional control, EMSA analysis using PmrA protein and its cognate *pmrA* promoter was conducted. PmrA bound exclusively to its own promoter and exhibited no interaction with the *trxB* promoter probes, demonstrating the specificity of the OxyR-*trxB* promoter interaction (Fig. 6D). Taken together, these findings demonstrate that OxyR directly binds to the *trxB* promoter. The specificity of the OxyR-*trxB* promoter interaction and its absence in the Δ*oxyR* mutant further establish the role of OxyR as a key transcriptional regulator of *trxB* in *F. tularensis* LVS.

### The Δ*trxB* mutant is attenuated for intramacrophage survival, and its clearance is dependent on ROS

**Fig 7:**
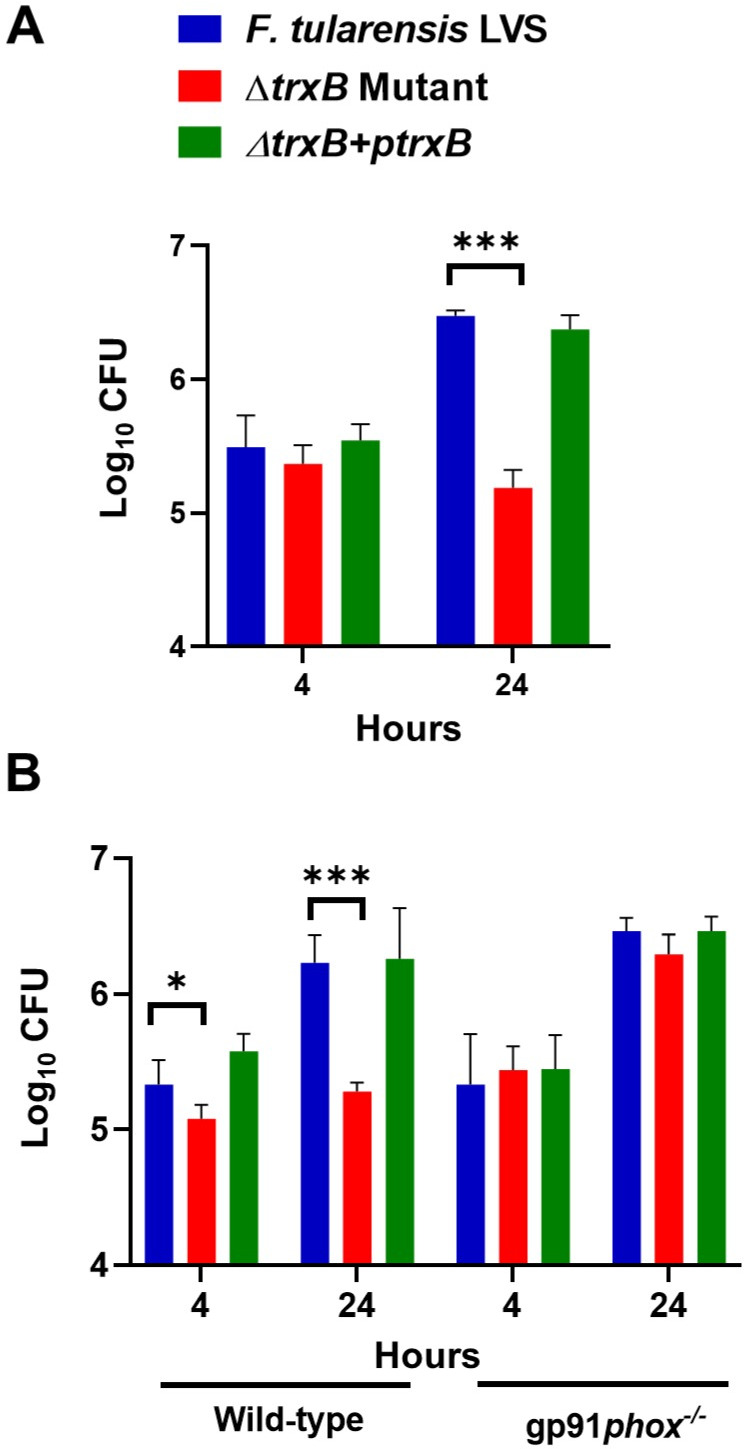
The Δ*trxB* mutant is attenuated for intramacrophage survival, and its clearance is dependent on reactive oxygen species. Murine macrophage cell line RAW264.7 (**A**) or primary BMDMs isolated from wild-type C57BL/6 mice or gp91*Phox*^−/−^ (**B**) were infected with the *F. tularensis* LVS, the Δ*trxB* mutant, or the transcomplemented strain (Δ*trxB* + p*trxB*) at 100 MOI of infection. The intramacrophage growth was quantified at 4 and 24 h post-infection. The results are expressed as log_10_ CFU/mL. The data represented as mean ± SEM are the cumulative results of three independent experiments, each conducted with four technical replicates and analyzed using one-way ANOVA. ***P < 0.001.

We performed a macrophage invasion assay in murine macrophage cell line RAW264.7 to determine the role of TrxB in macrophage survival. Almost equal numbers of WT *F. tularensis* LVS, the Δ*trxB* mutant, and the transcomplemented bacteria were recovered from the infected macrophages after 4 h of infection. However, nearly 100-fold less Δ*trxB* mutant as compared to the WT *F. tularensis* LVS and the transcomplemented bacteria recovered at 24 h post-infection (Fig. 7A). In primary bone marrow-derived macrophages (BMDMs) derived from WT C57BL/6 mice, the numbers of Δ*trxB* mutant bacteria recovered at 4 and 24 h post-infection were significantly lower than those observed for the WT *F. tularensis* LVS or the transcomplemented strain. However, the Δ*trxB* mutant bacteria replicated similarly to the WT *F. tularensis* LVS and the transcomplemented strain when the assays were conducted using *gp91phox*^−/−^ BMDMs that cannot generate the ROS (Fig. 7B). Collectively, these results demonstrate that TrxB contributes to the intramacrophage survival of *Francisella* by overcoming ROS-induced oxidative stress.

## DISCUSSION

TrxRs are NADPH-dependent enzymes that maintain redox homeostasis and contribute to bacterial virulence. These enzymes are essential for bacterial adaptation to oxidative stress, such as ROS generated during aerobic growth or by immune responses (34). In *M. tuberculosis*, the TrxR designated as TrxB2 is required to resist thiol-oxidizing stress and establish and maintain infection in mice (35). In *H. pylori*, TrxR regulates the redox state of proteins necessary for colonization and immune evasion (36). In *E. coli* and *S. aureus*, TrxRs regulate enzymes essential for bacterial survival under oxidative stress (37, 38). In *Clostridiodes difficile* and *Listeria monocytogenes*, TrxRs are involved in regulating virulence and repairing oxidative damage (39, 40). In this study, we demonstrate that the TrxR denoted as TrxB of *F. tularensis* LVS is critical for resistance to oxidative stress, antibiotics, and intramacrophage survival. Additionally, it reveals the complex antioxidative stress resistance mechanisms in *F. tularensis* and how their regulation systems are interconnected.

The amino acid sequence alignment revealed that TrxRs exhibit conserved functional motifs that are essential for their enzymatic activity and are consistently found across various bacterial pathogens, including *F. tularensis*. The most notable motif is the active site containing the conserved sequence CXXC, where the cysteine residues facilitate thiol-disulfide exchange reactions critical for reducing oxidized thioredoxin. Another key motif is the flavin adenine dinucleotide-binding domain, which supports the transfer of electrons from NADPH to the active site cysteines (22). The presence of these conserved features across diverse bacterial species highlights the role of TrxR in maintaining cellular redox balance and adapting to oxidative stress.

The results from this study demonstrate that the Δ*trxB* mutant of *F. tularensis* LVS is sensitive to oxidative stress induced by H_2_O_2_ and superoxide-generating compounds, paraquat and pyrogallol. These oxidants generate ROS that oxidize thiol groups in proteins, leading to the formation of disulfide bonds and disruption of cellular functions. Our previous studies have shown that *F. tularensis* encodes two thioredoxins, TrxA1 and TrxA2. However, the bacterium primarily relies on TrxA1 to counter oxidative stress and ensure intracellular survival (23). For H_2_O_2_, thioredoxins work in conjunction with peroxiredoxins such as AhpC to detoxify the oxidant, converting it into water and protecting cellular components from oxidative damage. TrxRs then catalyze the reduction of oxidized thioredoxins, restoring their ability to reduce disulfide bonds in proteins and repair oxidative damage (41). In the case of paraquat, which generates superoxide radicals, TrxRs indirectly mitigate oxidative stress by maintaining the reduced state of thioredoxin, thereby supporting the activity of antioxidant enzymes like superoxide dismutases (42). Pyrogallol, another ROS generator, induces oxidative stress by producing hydroxyl radicals. Similarly, the oxidative stress induced by diamide, a thiol-specific oxidizing agent, is countered by TrxRs through their role in maintaining the reduced state of thioredoxin (23). Diamide promotes the formation of disulfide bonds in proteins, leading to the oxidation of thiol groups and disruption of cellular redox homeostasis. In bacterial pathogens, TrxRs are upregulated in response to diamide-induced stress, ensuring efficient detoxification of reactive disulfides and supporting survival under oxidative stress conditions (43–45). Thus, TrxRs counter oxidative stress induced by oxidants by maintaining the reduced state of thioredoxin, which is essential for cellular redox homeostasis. The loss of TrxRs results in increased sensitivity to oxidants, as has been observed for the Δ*trxB* mutant of *F. tularensis* in this study.

The functional contributions of TrxRs to antibiotic resistance are not uniform across bacterial species and may vary depending on the antibiotic in question and the specific bacterial strain (46). Many antibiotics exert their antibacterial activity by inducing oxidative stress through the generation of ROS, particularly hydroxyl radicals, which contribute to bacterial killing (30). While TrxRs themselves do not directly confer antibiotic resistance, their role in regulating the redox state of enzymes involved in antibiotic metabolism or target binding has been implicated in resistance mechanisms in certain bacterial pathogens. TrxRs have been associated with metronidazole resistance in *H. pylori*, as they modulate the activity of redox-sensitive enzymes required for the bioactivation of metronidazole (47). In *M. tuberculosis*, TrxRs have been linked to resistance against streptomycin, potentially by influencing redox-sensitive pathways critical for drug efficacy (48). Furthermore, TrxRs have been shown to play a role in rifampicin resistance in *S. aureus*, possibly by modulating redox-dependent mechanisms affecting drug binding or metabolism (49). TrxRs also play a role in antibiotic resistance by influencing redox-sensitive enzymes involved in antibiotic detoxification or binding, as seen in species such as *P. aeruginosa* (50). Our results demonstrate that the loss of the *trxB* gene alters susceptibility to specific antibiotics, potentially by impacting the bacterial redox status. Specifically, ciprofloxacin, levofloxacin, and nalidixic acid, which showed significant differences in susceptibility, are known to trigger ROS production (30). These findings highlight the role of TrxB in mediating resistance against select antibiotics in *F. tularensis*.

The sensitivity of several bacteria to a gold-containing compound, auranofin, has been particularly of interest in the context of TrxRs. It has been reported that auranofin specifically targets TrxR in bacterial cells and inhibits its activity. The strains lacking TrxR are resistant to it, while the WT bacteria still retain their sensitivity. Auranofin has antibacterial activity against several bacterial species, including *S. aureus*, *S. pneumoniae*, and *M. tuberculosis* (31, 32, 51). Initially identified as a TrxR inhibitor due to its ability to disrupt cellular redox balance and induce oxidative stress, auranofin binds to the active site of TrxR, inhibiting its enzymatic activity (32). However, our results contrast with previous findings, demonstrating that the WT *F. tularensis* LVS and the ∆*trxB* mutant exhibit no differences in sensitivity to auranofin across various concentrations. These observations align with studies indicating that bacterial TrxRs are not direct targets of auranofin (52). Resistance or reduced susceptibility to auranofin in several gram-negative bacteria has been attributed to outer membrane proteins functioning as barriers, as well as the efficient efflux of the drug via multidrug efflux pumps. Additionally, an effective glutathione system has been proposed as a mechanism for auranofin resistance (52). Given that *Francisella* possesses all three structural features, its lack of susceptibility to auranofin may stem from any or all of these protective elements.

Our results demonstrate that the expression of primary antioxidant genes *ahpC*, *KatG*, and *sodB* was significantly reduced in the Δ*trxB* mutant when exposed to oxidative stress induced by H_2_O_2_ or diamide. We have reported that the expression of these primary antioxidant genes is controlled by OxyR, which is the key regulator of oxidative stress response in *F. tularensis* (16). OxyR is activated through the formation of a disulfide bond between conserved cysteine residues. Once activated, OxyR binds to the promoter regions of target genes and induces their transcription. This upregulation enhances the cellular capacity to counteract oxidative damage by maintaining redox homeostasis. The result from this study demonstrates that the expression of TrxB is regulated by OxyR under the conditions of oxidative stress.

The reciprocal downregulation of *oxyR* in the ∆*trxB* mutant and *trxB* in the ∆*oxyR* mutant suggests the presence of a redox-sensitive feedback loop that modulates oxidative stress responses in *F. tularensis*. Specifically, the decreased expression of *oxyR* in the ∆*trxB* mutant under oxidative stress, along with evidence that OxyR directly regulates *trxB* transcription, implies a regulatory circuit in which TrxB functions not only as a downstream effector of OxyR but also as a contributor to OxyR activity. Our previous studies demonstrated that *oxyR* expression is regulated by thioredoxin TrxA1 under oxidative stress conditions in *F. tularensis* (23). This supports the existence of a redox-sensitive feedback mechanism wherein oxidized TrxA1 promotes *oxyR* expression, OxyR induces *trxB,* and TrxB subsequently reduces TrxA1, thereby sustaining its regulatory function. Such an interdependent loop may ensure tight control of the oxidative stress response, enabling *Francisella* to rapidly adapt to fluctuating redox conditions. A similar redox-sensitive regulatory feedback loop has been documented in *E. coli* and *P. aeruginosa*, where OxyR transcriptionally activates antioxidant enzyme genes such as *katG* and *ahpCF*, while the thioredoxin and glutaredoxin systems modulate the redox state of OxyR itself (53, 54).

Our findings demonstrate that TrxB in *F. tularensis* LVS contributes to intracellular survival, primarily by counteracting ROS-mediated oxidative stress. The significant reduction in *∆trxB* mutant recovery at 24 h post-infection in RAW264.7 macrophages and WT C57BL/6 BMDMs highlights its critical function in oxidative stress resistance. However, in *gp91phox*^−/−^ BMDMs, which lack NADPH oxidase-dependent ROS generation, the *∆trxB* mutant replicated similarly to WT and transcomplemented strains, confirming that TrxB specifically mitigates oxidative damage induced by macrophage-derived ROS.

Although not investigated in this study, the Type A (*F. tularensis* subspecies *tularensis*) and Type B (*F. tularensis* subspecies *holarctica*) strains are more virulent than *F. tularensis* LVS. Given the role of TrxB in oxidative stress responses in *F. tularensis* LVS, it is plausible that similar mechanisms operate in these strains, allowing them to evade host defenses and establish infection. Further investigation into the thioredoxin system of human virulent Type A and B strains could provide insights into their enhanced virulence and potential therapeutic targets. To conclude, this study describes the role of an important uncharacterized component of the oxidative stress response machinery of *F. tularensis* LVS required for resistance to oxidative stress and intramacrophage survival.

## MATERIALS AND METHODS

### Bacterial strains and culture media

The bacterial strains, plasmids, and mutants generated in this study are listed in Table 2. The *F. tularensis* subspecies *holarctica* LVS obtained from the American Type Culture Collection, Rockville, MD (ATCC 29684) was used in this study. The deletion mutant of the master regulator of the oxidative stress response (Δo*xyR*) and its transcomplemented strain (Δo*xyR* + p*oxyR*) has been previously generated and characterized in our laboratory (16). The *F. tularensis* cultures were grown on Mueller-Hinton (MH) chocolate agar plates supplemented with IsoVitaleX at 37°C with 5% CO_2_; MH broth supplemented with ferric pyrophosphate and IsoVitaleX (BD Biosciences, San Jose, CA) at 37°C with shaking (160 rpm). Active mid-log phase bacteria grown in MH-broth were harvested and stored at −80°C. One milliliter aliquots was thawed periodically for use. *E. coli* strain DH5α was used for routine cloning. *E. coli* cultures were grown in Luria-Bertani (LB) broth or on LB agar plates. When necessary, kanamycin (25 µg/mL) or hygromycin (200 µg/mL) was included in broth and agar media for selection purposes.

**TABLE 2:** List of bacterial strains, plasmid vectors, and primers used in this study

*Francisella* strains	Genotype	Source
*F. tularensis* LVS	Wild-type strain	ATCC
MF024	*F. tularensis* LVS. RpoC-VSVG fusion, Kan^r^	(16)
MF025	*F. tularensis* LVS. OxyR-VSVG fusion, Kan^r^	(16)
MF042	*F. tularensis* LVS *trxB* gene deletion mutant (∆*trxB*)	This study
MF043	*F. tularensis* LVS, ∆*trxB,* pMM025(pMP822 + *trxB*), Hygro^r^	This study
*E. coli Strains*		
DH5α	F– Φ80*lac*ZΔM15 Δ(*lac*ZYA-*arg*F) U169 *rec*A1 *end*A1 *hsd*R17 (rK–, mK+) *pho*A *sup*E44 λ– *thi*-1 *gyr*A96 *rel*A1	Invitrogen
Plasmids		
pMP822	*E. coli-Francisella* shuttle vector, Hygro^r^	(28, 29)
pJC84	*E. coli-Francisella* suicide vector, Kan^r^	(27)
pMM024	pJC84+ fused flanking fragment of *trxB* gene, Kan^r^	This study
pMM025	pMP822 + *trxB,* Hygro^r^	This Study

Primers	Sequence	Purpose
∆*trxB* deletion		
*trxB* upstream fragment		
MP625	5′-CAAggatcctgaact taatcatgag ctaggagt-3′	F-primer with a *BamH*I site
MP626	5′-gcCATtttttttaaaaaatttttga-3′	R-primer
*trxB* downstream fragment		
MP627	5′-caaaaattttttaaaaaaaATGgctcaaTAAaatataaaccctcactt-3′	F-primer
MP628	5′-TGATgtcgactcgg ttctgtgcacaaaaatagtt-3′	R-primer with a *Sal*I site
*trxB* mutant screening and verification		
MP037	5′-CCGGATCCATGAAATTTGAATTACCAAAAC-3′	F-primer for *sodB* as control
MP038	5′-CGCTGCAGCTAATCAGCGAATTGCTCAGAAAC-3′	R-primer for *sodB* as control
MP629	5′-TGCTCATACATATTCGCAACCTGT-3′	F-primer Flanking *trxB*
MP630	5′-CATTTGCTAAACCATTGATATT-3′	R-primer Flanking *trxB*
MP641	5′-ttggtggtggcaacactgct-3′	F-primer Inside *trxB*
MP642	5′-tgatacgcagagcattaactccca-3′	R-primer Inside *trxB*
*trxB* complementation vector		
MP631	5′-CAAGGATCCATGGCAAATCATCATAAACTAA-3′	F-primer with a *BamH*I site
MP632	5′-CGACCTCGAGTTATTGATCTAAATTATCTAAA-3′	R-primer with an *Xho*I site
Transcriptional analysis		
*tul4* (Internal control)		
MP029	5′-TCGCAGGTTTAGCGAGCTGTTCTA-3′	F-primer
MP030	5′-ACAGCAGCAGCTTGCTCAGTAGTA-3′	R-primer
*katG (FTL_1504*)		
MP077	5′-CCTGCCAAATAAAGTTTTGCTC-3′	F-primer
MP078	5′-AGCTCACCAATGGACTCCTAC-3′	R-primer
*sodB* (*FTL_1791*)		
MP101	5′-GGCGGAATATTTAATAACGCTGC-3′	F-primer
MP102	5′-GTGCTCCCAAACATCAAAAG-3′	R-primer
*ahpC* (*FTL_1015*)		
MP258	5′-TTGTATTCTCATTACCAGGAGCA-3′’	F-primer
MP259	5′-ACAATCATTGCATAGCGCCA-3′	R-primer
*oxyR* (*FTL_1014*)		
MP528	5′-ATGCCTAAAATTCTCCCTGC-3′	F-primer
MP529	5′-GACCTTCATCAAGAAGCATAAGA-3′	R-primer
t*rxA2* (*FTL_1224*)		
MP286	5′-CAGACGAAGCTAATTTTGACAAAC-3′	F-primer
MP287	5′-AGTCTCAAGTTGCTTACGATTTTT-3′	R-primer
*trxA1* (*FTL_0611*)		
MP536	5′-GTGGTCCTTGCAAAATGCTT-3′	F-primer
MP537	5′-CACGTACACCAAATTTCATCGC-3	R-primer
*trxB*(*FTL_1571*)		
MF530	5′-GAAGTTTATGGGTAAAGGTGTCTC-3′	F-primer
MF531	5′-TCCCATATCATCGCCAAGAA-3′	R-primer
ChIP analysis (qPCR) and EMSA for *trxB* locus		
MP358	5′-TGCTGGTTGGGCAAATCTA-3′	F-primer for *fopA* coding region
MP359	5′-TGTAGTCGCACCATTATCCTGA-3′	R-primer for *fopA* coding region
MP384	5′-TTTGAACTTAATCATGAGCTAGGA-3′	F-primer for ChIP region 1
MP385	5′-TTCCAGATACAATCATTACTTGGA-3	R-primer for ChIP region 1
MP386	5′-catattcgcaacctgttggt-3′	F-primer for ChIP region 2
MP386b	5′-Biotin-catattcgcaacctgttggt-3′	F-primer for EMSA probe
MP387	5′-gcaggcccagatcctaaaatta-3′	R-primer for ChIP region 2
MP388	5′-gtgctacttgtgatggcttctt-3′	F-primer for ChIP region 3
MP389	5′-aactcccatatcatcgccaa-3′	R-primer for ChIP region 3
*pmrA* EMSA		
MP402	5′-GGGAGCTAGTGAGAGGAATTTTT-3′	F-primer for *pmrA* promoter
MP402b	5′-Biotin-GGGAGCTAGTGAGAGGAATTTTT-3′	F-primer for EMSA probe
MP403	5′-CCAAATGAAGATCATCTTCAGCC-3′	R-primer for *pmrA* promoter

### Bioinformatics analysis

Sequences of TrxR from *F. tularensis* LVS and other bacterial species were obtained from the National Center for Biotechnology Information. Using Clustal Omega, multiple sequence alignments were performed to determine both conserved and diverse features of TrxR across these bacterial species. TrxRs from three subspecies *F*. *tularensis* subspecies *holarctica* LVS (*FTL_1571, trxB*), *F. tularensis* subspecies *tularensis* SchuS4 (*FTT_0489*c, *trxB*), and *F. novicida* (FTN_0580*, trxB*) were aligned. TrxRs from *E. coli* (*b0888, trxB*), *S*. Typhi (*t1976, trxB*), *Y. pestis* (*YPO1374, trxB*), *H. pylori* (*HP_0825, trxB*), *P. aeruginosa* (*PA2616, trxB1*), *S. aureus* (*SACOL0829, trxB*), *B. subtilis* (*BSU_34790, trxB*), *M. tuberculosis* (*Rv3913, trxB1/trxR*), and *V. cholerae* (*Vch1786_I0686, trxB*) were also included in the analysis.

### Construction of *F. tularensis* LVS *trxB* (*FTL_1571*) gene deletion mutant (∆*trxB*) and transcomplemented (∆*trxB* + p*trxB*) strains

A gene deletion mutant of the *trxB* gene (∆*trxB*) in *F. tularensis* LVS was constructed by deleting the entire coding region of the *trxB* gene, using the SacB-assisted allelic replacement methods described earlier (26, 27). To create the ∆*trxB* mutant, 850 base pairs (bp) upstream and 954 bp downstream fragments flanking the *trxB* gene were amplified by PCR using primers MP625/626a and MP627/628, respectively, and fused by overlap extension PCR. The resulting 1790 bp fragment was cloned into a suicide vector p*JC84,* at *BamH*I and *Sal*I sites, generating a plasmid p*MM024. F. tularensis* LVS was transformed with p*MM024* by electroporation, as described earlier (16). Following transformation, kanamycin-resistant clones were selected on MH-chocolate agar plates containing 10 µg/mL kanamycin. The kanamycin-resistant clones were plated on MH-chocolate agar plates containing 8% sucrose, and the kanamycin-sensitive clones were selected. These clones were screened for allelic replacement by PCR with *trxB* gene-specific primers and confirmed through a duplex PCR using *sodB* gene primers as internal controls, followed by DNA sequencing. To complement the *F. tularensis ∆trxB* mutant, the *trxB* gene sequence (*FTL_1571*) was PCR amplified using primers MP631 and MP632. The amplified fragment was then digested with *BamH*I and *Xho*I restriction enzymes and cloned into the *E. coli-Francisella* shuttle vector p*MP822* (29). The resulting plasmid, p*MM025*, was verified through PCR and DNA sequencing. Subsequently, p*MM025* was transformed into the *∆trxB* mutant by electroporation, and transformants were selected on MH-chocolate agar plates containing 200 µg/mL hygromycin. PCR analysis confirmed the successful complementation of the mutant strain.

### Testing the susceptibility of *∆trxB* mutant of *F. tularensis* LVS to oxidants

The WT *F. tularensis* LVS, the *∆trxB* mutant, and the transcomplemented strains were tested for susceptibility to H_2_O_2_, diamide, paraquat, and pyrogallol using growth curves and bacterial killing assays. For growth curves, bacterial suspensions (OD_600_ = 0.05) were incubated in MH-broth with or without 1 mM H_2_O_2_ at 37°C with constant shaking, and OD_600_ values were recorded every four hours for 28 h. In bacterial killing assays, bacterial suspensions (1 × 10⁹ CFU/mL) were exposed to 0.5, 1, and 1.5 mM of H_2_O_2_ or 10, 50, and 250 µM of diamide at 37°C. Similar killing assays were conducted using superoxide-generating compounds paraquat (1 mM) and pyrogallol (1 mM). Samples were taken at 1, 2, and 3 h, serially diluted, and plated on MH-chocolate agar plates. The plates were incubated at 37°C in the presence of 5% CO_2_, and the colonies were counted 48 h later. The bacterial counts were expressed as log_10_ CFU/mL.

### Antibiotic sensitivity assays

The sensitivity of WT *F. tularensis* LVS, the Δ*trxB* mutant, or the transcomplemented strain to several antibiotics was determined using disc diffusion assays. The bacterial cultures grown on MH chocolate agar plates were scraped and suspended in MH-broth to achieve an OD_600_ of 1.0. The bacterial suspensions were spread with a sterile cotton swab onto MH chocolate agar plates. Sterile antibiotic discs were placed on the plates. The plates were incubated at 37°C in the presence of 5% CO_2_. After 3 days of incubation, the zones of growth inhibition around the disks were measured. The experiments were repeated at least three times for each antibiotic and the bacterial strain.

Both growth curves and killing assays were also performed to test the sensitivity of the WT *F. tularensis* LVS, the Δ*trxB* mutant, or the transcomplemented strain to auranofin. For growth curves, bacterial suspensions (OD_600_ = 0.05) were incubated in MH-broth with or without 1, 5, 10, and 20 mM auranofin at 37°C with constant shaking, and OD_600_ values were recorded every 4 h for 28 h. In bacterial killing assays, bacterial suspensions (1 × 10⁹ CFU/mL) were exposed to similar concentrations of auranofin at 37°C. Samples were taken at 1, 3, and 6 h, serially diluted, and plated on MH-chocolate agar plates. The plates were incubated at 37°C in the presence of 5% CO_2_ and the colonies were counted 48 h later. The bacterial counts were expressed as log_10_ CFU/mL.

### Analysis of gene expression

Overnight cultures of WT *F. tularensis* LVS, the *∆trxB* mutant, and the transcomplemented strain were adjusted to an OD_600_ of 0.2 and grown for 2 h at 37°C with shaking in 10 mL of MH-broth, both in the absence or presence of 1 mM H_2_O_2_ or 50 µM diamide. Similarly, in another experiment, WT *F. tularensis* LVS, the Δ*oxyR* mutant, and Δ*oxyR* + p*oxyR* transcomplemented strains were either left untreated or treated with 1 mM H_2_O_2_. Total RNA was extracted from the grown cultures using Purelink RNA Mini Kits (Ambion) and further treated with DNase to eliminate contaminating DNA. cDNA synthesis was performed using the iScript cDNA Synthesis Kit (Ambion). Quantitative PCR (qPCR) was then conducted with iQ SYBR Green Supermix (Bio-Rad) to analyze the expression of target genes, using the *tul4* gene of *F. tularensis* LVS as an internal control. Relative gene expression was calculated using the 2−ΔΔCT method: 2− [ΔCT (mutant) − ΔCT (WT)], where ΔCT = CT of the target gene − CT of the internal control. Data were presented as the mean ± SD of three biological replicates. The primer sequences of the target genes used for qPCR are detailed in Table 2.

### Western blot analysis

Overnight cultures of WT *F. tularensis* LVS, the *∆trxB* mutant, and the transcomplemented strain were adjusted to an OD_600_ of 0.2 and grown for 2 h at 37°C with shaking in 10 mL MH broth in the absence or presence of 1 mM H_2_O_2_ or 50 µM diamide. Bacterial cultures were harvested, centrifuged, and re-suspended in 200 µL of lysis buffer containing 200 mM Tris-HCl (pH 8.0), 320 mM (NH_4_)_2_SO_4_, 5 mM MgCl_2_, 10 mM EDTA, 10 mM EGTA, 20% glycerol, 1 mM dithiothreitol (DTT), and protease and phosphatase inhibitors. Protein concentrations of cell lysates were determined using the Bio-Rad Protein Assay. Five micrograms of protein from each sample was separated on a 12% SDS-PAGE gel, transferred to polyvinylidene difluoride membranes (Millipore), and probed with primary antibodies against KatG (1:20,000) and SodB (1:20,000). Secondary anti-rabbit antibodies conjugated to horseradish peroxidase (HRP) (Santa Cruz; 1:10,000) were used for detection. Protein bands were visualized using Supersignal West Pico chemiluminescent substrate (Thermo Scientific) and imaged on a Chemidoc XRS system (Bio-Rad). For loading control, the membranes were stripped and re-probed with anti-FopA antibodies. The anti-SodB, KatG, and FopA antibodies were kindly provided by Dr. Karsten Hazlett (Albany Medical College, Albany, NY).

### ChIP assays

To study the binding activity of OxyR *in vivo* to the promoter region of the *trxB* gene, an *F. tularensis* LVS OxyR-VSVG strain expressing *F. tularensis* LVS OxyR protein fused with the C-terminal VSVG tag was constructed as described earlier (16). The tagging integration vector, p*KL02*, encoding an RpoC-VSVG tag protein, kindly provided by Dr. Simon Dove (Boston Children’s Hospital Division of Infectious Diseases, Boston, MA), was used as a positive control. *F. tularensis* LVS-OxyR-VSVG and RpoC-VSVG tag strains were cultured in 50 mL MH-broth at 37°C with constant shaking. At an OD_600_ of approximately 0.4, rifampicin (50 µg/mL; Sigma) was added to the RpoC-VSVG cultures for 30 min before crosslinking. All cultures were then crosslinked with 1% formaldehyde for 30 min, followed by quenching with 250 mM glycine for 5 min. Cells were washed three times with 1 × PBS, resuspended in lysis buffer containing protease inhibitors (Sigma), and sonicated to lyse cells and shear chromosomal DNA to fragments of ~500 bp. Cell debris was removed by centrifugation, and the supernatants were adjusted for salt concentration. Immunoprecipitation was performed overnight at 4°C using anti-VSVG agarose beads (Sigma). A 50 µL aliquot of the supernatant was diluted in 200 µL TE + 1% SDS to be used as an input control. Immunoprecipitates were washed five times with IPP150 buffer, two times with TE buffer, and eluted using 150 µL elution buffer and 100 µL TE + 1% SDS buffer, respectively (16). Elutes and input samples were incubated overnight at 65°C to reverse crosslinking, and the DNA was purified using a PCR Purification Kit (Qiagen). ChIP and the input DNA samples were analyzed by qPCR to determine the proportion of specific DNA fragments. qPCR values were normalized to inputs using an internal *fopA* coding region control. ChIP assays using WT *F. tularensis* LVS (mock) and rpoC-VSVG (positive control) strains were included for comparison. Results were expressed as relative enrichments of the detected fragments. The primer sequences used for qPCR are detailed in Table 2.

### EMSA

The EMSA was performed using bacterial lysates and the LightShift Chemiluminescent EMSA Kit (Thermo Scientific) as reported earlier (16). Briefly, the bacterial lysates were prepared from bacterial cultures grown similar to those described above for the gene transcriptional analysis. Promoter DNA probes were generated from *F. tularensis* LVS genomic DNA by PCR using a biotin-labeled 5′ forward primer (Integrated DNA Technologies) and an unlabeled reverse primer. Competitor DNA was amplified using the same unlabeled primer pair, and both probes and competitors were purified with a PCR purification kit (Invitrogen). Primer sequences MP386/MP387 (Table 2) for promoter regions of the *trxB* gene were designed to amplify an 182 bp fragment from −138 to +43 relative to the *trxB* open reading frame. Primer sequences MP402/MP403 (Table 2) used for the *pmrA* promoter region were designed to amplify a 505 bp upstream intergenic region. Biotin-labeled primers MP386b and MP402b (Table 2) were used for probe generation. The binding of the transcriptional regulator PmrA to its promoter served as a positive control to validate protein activity in the Δ*oxyR* mutant lysates. Protein extracts were prepared from *F. tularensis* LVS, the Δ*oxyR* mutant, and transcomplemented strains grown to OD_600_ of 0.5 in MH-broth at 37°C. After harvesting and washing, the cells were resuspended in TE buffer (10 mM Tris, pH 7.4; 1 mM EDTA, pH 8.0) with protease inhibitors (Sigma). The cells were lysed by sonication, and the soluble protein fraction was separated by centrifugation for 15 min (4,000 × *g*) at 4°C. The protein concentrations were determined using a Bio-Rad Protein Assay. For binding reactions, 1 ng of DNA probe was incubated with 5 µg of total protein in 20 µL reaction buffer following EMSA kit instructions. Competitor DNA (30 ng) was added as indicated. Reactions were loaded onto a 5% TBE non-denaturing gel (Bio-Rad), electrophoresed in 0.5 × TBE buffer, and transferred to Hybond-N + nylon membranes (Amersham). Membranes were crosslinked and probed with streptavidin-HRP conjugates to detect biotin-labeled DNA. A chemiluminescent substrate was used for visualization, and DNA bands were imaged using a Chemidoc XRS system (Bio-Rad).

### BMDM isolation and culture

BMDMs were derived from WT and *gp91phox*^−/−^ mice using standard isolation protocol (55) approved by the Institutional Animal Care and Use Committee of Albany College of Pharmacy and Health Sciences, Albany, New York. Mice were anesthetized and euthanized before femur dissection. Bone marrow was flushed with ice-cold DMEM, centrifuged, and resuspended in a complete BMDM medium. Monocytes were cultured for seven days in 5% CO_2_ at 37°C, allowing differentiation into adherent macrophages. The cells were harvested using ice-cold PBS, scraped, and resuspended in the medium for experimental use or cryopreservation in FBS with 10% DMSO. BMDMs were thawed, resuspended in medium, and counted using a hemocytometer and Trypan Blue exclusion assay. The cells were seeded in 6-, 12-, or 24-well plates and incubated at 37°C with 5% CO_2_ overnight prior to use in macrophage invasion assays.

### Macrophage invasion assay

To evaluate the intracellular survival of WT *F. tularensis* LVS, the *∆trxB* mutant, and the transcomplemented strain, a gentamicin protection assay was conducted as described earlier (56). Briefly, the murine macrophage cell line RAW264.7 or BMDMs isolated from C57BL/6 mice were infected with *F. tularensis* LVS, the *∆trxB* mutant, and the transcomplemented strains at a multiplicity of infection of 100. Two hours post-infection, macrophages were treated with gentamicin (100 µg/mL) for 2 h to eliminate the extracellular bacteria. The gentamicin-containing medium was then replaced with an antibiotic-free medium, followed by incubation at 37°C in 5% CO_2_. Macrophages were harvested at 4 and 24 h post-infection and lysed using 0.1% sodium deoxycholate. The lysates were serially diluted in sterile PBS and plated on MH-chocolate agar plates for bacterial enumeration. Results were expressed as log_10_ CFU/mL.

### Statistical analysis

All data were statistically analyzed using one-way ANOVA followed by Tukey-Kramer Multiple Comparison tests or the Student’s *t*-test and were expressed as means ± SEM or SD. The value of *P* < 0.05 was considered statistically significant.

## SUPPLEMENTAL MATERIAL

Figure S1 jb.00173-25-s0001.tiff Confirmation of trxB gene deletion, transcomplementation, and bioinformatic analysis.

ASM does not own the copyrights to Supplemental Material that may be linked to, or accessed through, an article. The authors have granted ASM a non-exclusive, world-wide license to publish the Supplemental Material files. Please contact the corresponding author directly for reuse.
