# Supplementary figures and images for: Role of thioredoxin reductase (TrxB) in oxidative stress response of Francisella tularensis live vaccine strain
Source: J Bacteriol. 2025 Sep 3;207(10):e00173-25. doi: 10.1128/jb.00173-25 (PMC12478593; doi:10.1128/jb.00173-25)

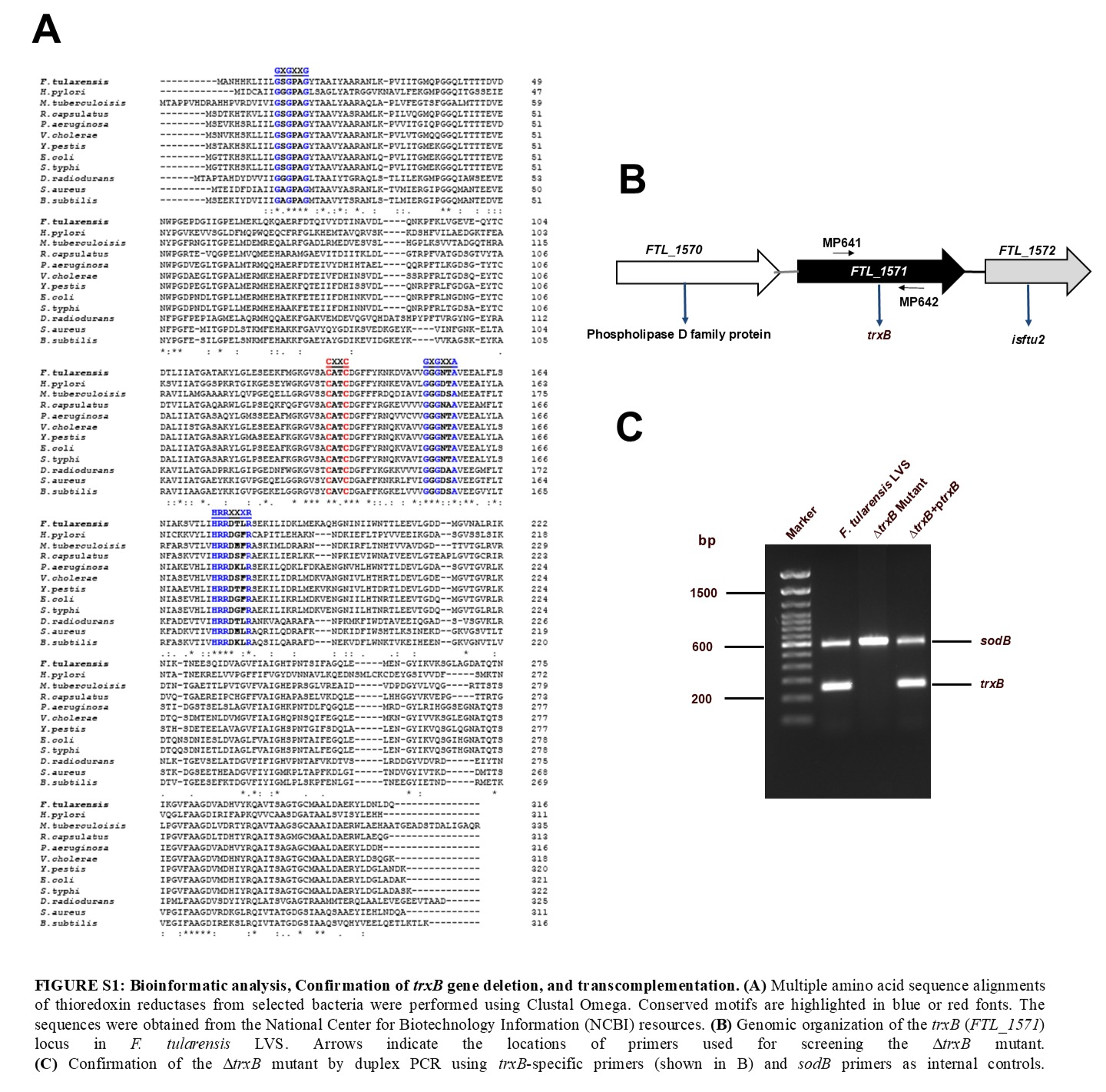

Supplement: Figure S1 — Confirmation of trxB gene deletion, transcomplementation, and bioinformatic analysis. [file jb.00173-25-s0001.tiff]
